# Redefining Antifungal Treatment: The Innovation of Metal-Based Compounds

**DOI:** 10.1021/acs.jmedchem.4c02084

**Published:** 2025-02-21

**Authors:** Joana Cardoso, Eugénia Pinto, Emília Sousa, Diana I. S. P. Resende

**Affiliations:** † Laboratory of Organic and Pharmaceutical Chemistry, Faculty of Pharmacy of the University of Porto, 4050-313 Porto, Portugal; ‡ Laboratory of Microbiology, Faculty of Pharmacy of the University of Porto, 4050-313 Porto, Portugal; § Interdisciplinary Centre of Marine and Environmental Research (CIIMAR), University of Porto, 4450-208 Matosinhos, Portugal; ∥ Department of Chemistry, School of Medicine and Biomedical Sciences (ICBAS), University of Porto, 4050-313 Porto, Portugal

## Abstract

Fungal infections are a significant contributor to global morbidity and mortality, particularly among immunocompromised patients. With the increasing prevalence of drug-resistant strains, it has become imperative to identify alternative approaches. Metal ion coordination enhances drug efficacy through novel modes of action and may hinder resistance mechanisms. This article aims to identify gaps in the current metal-based antifungal therapy to guide research directions and facilitate drug development. Relevant metal complexes, together with their ligands, have been categorized according to their metal coordination and their activities highlighted. Most examples reported were found to be more effective against drug-resistant strains than non-coordinated ligands, thus establishing the importance of metal ion and co-ligand(s) nature, the influence of electron-withdrawing substituents on structure–activity relationships, and the unique structural features of Schiff bases. Although still at the preclinical phase, the *in vitro* efficacy of these examples suggests that metal-based drugs may represent a promising approach to overcoming antifungal resistance.

## Graphical abstract

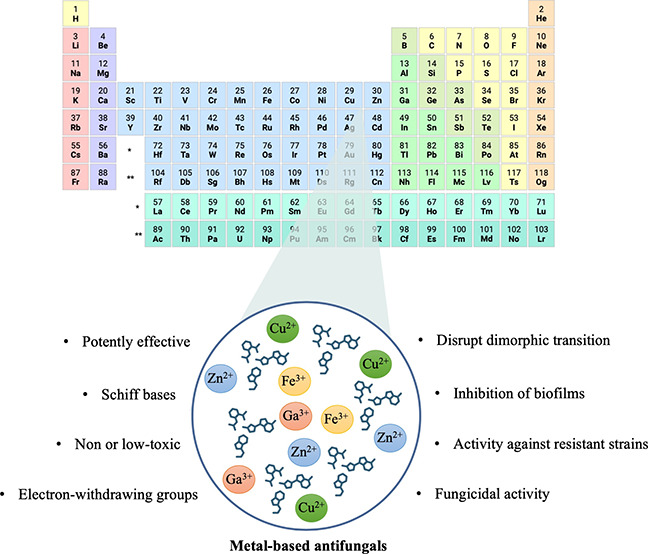

## Significance

- This Perspective explores the potential of metal-based compounds as innovative candidates for antifungal therapy, particularly against drug-resistant strains.
- Offers a comprehensive analysis of critical factors, including structural features, to guide research and accelerate drug development.
- Highlights the role of metal ion coordination in enhancing antifungal efficacy through diverse mechanisms of action, aiming to mitigate resistance development.

## 1 Introduction

The incidence and severity of fungal infections have increased dramatically in recent decades and have become a major cause of mortality and morbidity.^1^ This growing problem is attributable to the emergence of resistance to traditional antifungal agents and the increase in the immunocompromised population who are particularly susceptible to the development of systemic infections often associated with the genera *Candida*, *Aspergillus*, and *Cryptococcus*.^2,3^ Current therapeutic options for fungal infections are limites to five main classes: polyenes, azoles, echinocandins, flucytosine, and allylamines; each of these drug classes targets distinct cellular components and mechanisms of action. Despite the diversity of these classes, many of the available antifungal agents have suboptimal efficacy, limiting their utility in clinical practice.^4,5^ In light of this situation, there is an urgent clinical need for novel classes of drugs with antifungal properties and appropriate adjuvants.^6,7^ This approach will ensure that the therapeutic armamentarium is refreshed, thereby facilitating the selection of better treatment options for each patient and preventing the emergence of new resistances.^8^ Nevertheless, the development of a safe and broad-spectrum antifungal drug is an extremely complex process, especially due to the significant physiological parallels between human and fungal organisms.^9^ Therefore, the progress of new antifungals has been slower than that of new antibacterial agents. In recent years, numerous natural and synthetic compounds have reached advanced stages of clinical development;^10,11^ however, most candidates are structural analogues of previously approved agents acting on the same target, which poses a challenge for resistance resolution.

Metals such as gold, platinum, and titanium have historically played an important role in medicine, with therapeutic, diagnostic, medical devices, and equipment purposes.^12^ More recently, their significance has extended into drug discovery, facilitating the design and synthesis of novel therapeutic agents and thus becoming indispensable to the pharmaceutical industry.^13^ Transition metal complexes can offer diverse properties compared to their purely organic counterparts, as metal ions possess a wide range of coordination numbers and geometries, allowing access to multiple distinct modes of action when combined with different ligands.^14^ Several authors have reported that the coordination of ligands with metal ions can greatly enhance their biological activities, probably due to the greater lipophilicity of the formed complexes. It has been suggested that chelation reduces the polarity of the metal ion by partially sharing its positive charge with the donor groups. This, in turn, increases the lipophilicity of the complex, which favors its permeation into the lipid layer of the cell membrane and affects the growth and development of pathogenic microorganisms.^15^ Metal complexes stability constants are used to quantify the equilibrium of a metal–ligand complex in solution, reflecting the strength of the interaction between the metal ion and its ligands.^16^ These constants are determined through experimental methods, such as ultraviolet–visible (UV–Vis) spectroscopy, isothermal calorimetry, and fluorescence spectroscopy, which measure changes in chemical or physical properties during complex formation.^17^ Recently, artificial intelligence approaches, in particular machine learning, have become increasingly effective tools for predicting stability constants, as this can be achieved through the analysis of extensive data sets of structural, electronic, and thermodynamic parameters, thereby facilitating rapid and accurate predictions without the need for extensive experimentation.^18,19^ In medicinal chemistry, stability constants are crucial for understanding the behavior of metal complexes in biological systems. They have a profound influence on key properties, including bioavailability, selectivity and therapeutic efficacy. High stability constants are indicative of high metal–ligand affinity, which is essential to ensure the integrity of the complex under physiological conditions. This allows for targeted delivery, prolonged activity, and improved therapeutic outcomes, thus representing a cornerstone in the design of metal-based drugs.^20,21^

Metal-based antimicrobial therapy has demonstrated promising results in fighting the increasing threat of infections and has gained substantial attention in recent years. Salvarsan, an organoarsenic compound, is a remarkable example of an antibiotic drug; its success in the treatment of syphilis was instrumental in the development of modern metal-based antimicrobial chemotherapy.^22^ Metal ions have demonstrated intrinsic antimicrobial activity, with metal complexes having the potential for innovative modes of action that may be beneficial in the treatment of infections resistant to conventional antimicrobial agents.^23^ These exceptional properties of metal–ligand complexes make them an interesting starting point for the investigation of new antifungal drug options. While numerous comprehensive articles have detailed the use of metal-based compounds as antimicrobials,^24–26^ there is a lack of research exploring their potential as new antifungal drugs. This paucity of research is primarily attributable to the limited number of systematic studies conducted both *in vitro* and *in vivo*. Additionally, the existing literature on metal-based antifungals^14,27,28^ only offers overviews, failing to delve into important aspects regarding the nature of ligands and their complexes. Notably, this includes the crucial significance of establishing structure–activity relationships (SARs), as this is vital for elucidating the mechanism of action, optimizing antifungal efficacy, and guiding the rational design of new derivatives. Consequently, it is imperative to gain comprehensive insights into specific functionalities in order to facilitate the development and application of metal-based antifungal drugs in the future. Recent research has been dedicated to the synthesis and biological evaluation of azole-metal complexes, demonstrating that the coordination of azoles, a widely used class of antifungal agents, with transition metals enhances antifungal activity. In view of the extensive existing literature^29–34^ and comprehensive reviews^35–38^ on this topic, the present article does not intend to replicate these efforts by discussing azole-metal complexes. Instead, the present article concentrates on recent advances in the design of innovative metal-based compounds, with a particular emphasis on their potential for the treatment of fungal infections and the resolution of antifungal resistance issues. In order to achieve this objective, a selection of metal-based compounds was made on the basis of their relevant antifungal profiles, as reported over the preceding decade. An in-depth analysis of the fundamental aspects of these compounds was then conducted, including their antifungal activity against drug-resistant strains, SARs, cytotoxicity, and antivirulence mechanisms. The discussion that follows highlights the potential of metal-based drugs as a promising and innovative therapeutic strategy, offering more effective and alternative treatment options.

## 2 NEW METAL-BASED ANTIFUNGALS

Several studies have investigated the potential of metal complexes as antifungal agents, demonstrating their efficacy against a diverse range of fungal pathogens.^25^ Among the metals, silver, copper, manganese, zinc, gold, nickel, iron, and ruthenium have been the subject of the most extensive research due to their diverse physicochemical properties.^39^ Silver-based antifungal compounds have been widely studied for their broad-spectrum activity against a range of fungal species.^40^ A study by Stanković et al.^29^ demonstrated that silver­(I) complexes incorporating clinically used antifungal azoles—econazole (**ECZ**), voriconazole (**VCZ**), and clotrimazole (**CTZ**)—significantly improved antifungal activity against *Candida* spp. when compared to the azole drugs. Notably, [Ag­(**VCZ**)_2_] showed a remarkable 9533-fold increase in efficacy against *Candida glabrata* in comparison to its free ligand. Additionally, these complexes inhibited the hyphae and biofilm formation of *Candida albicans* at subinhibitory concentrations. [Ag­(**CTZ**)_2_] was also found to reduce the adhesion of *C. albicans* to human A549 cells, an essential step in fungal infection, and together with [Ag­(**VCZ**)_2_] reduced ergosterol levels in *C. albicans* at 0.5 × MIC. Notwithstanding the encouraging antifungal outcomes, the study found that the cytotoxicity of [Ag­(**ECZ**)_2_] and [Ag­(**VCZ**)_2_] against healthy human fibroblasts (MRC-5) was comparable to and higher, respectively than that of their free counterparts whereas [Ag­(**CTZ**)_2_] exhibited a more favorable toxicity profile compared to **CTZ**. The potential of gold-based compounds as antifungal agents has also been investigated, albeit to a lesser extent than that of silver-based compounds. Gold complexes, in particular those comprising gold­(I) and gold­(III) ions, have been demonstrated to exhibit notable antifungal activity. Specifically, the coordination of gold­(III) to azole drugs has been shown to enhance their antimicrobial properties.^30^ The complexes with clinically used antifungal azoles—**CTZ**, **ECZ**, tioconazole (**TCZ**), and **VCZ**—demonstrated markedly elevated anti-*Candida* activity in comparison to the uncoordinated azoles; [Au­(**TCZ**)­Cl_3_] and [Au­(**VCZ**)­Cl_3_] demonstrated the most substantial improvement. Furthermore, the study revealed that [Au­(**TCZ**)­Cl_3_] and [Au­(**ECZ**)­Cl_3_] were effective in reducing the production of pyocyanin, a virulence factor in *Pseudomonas aeruginosa*, a bacterium associated with respiratory infections.

Specifically, complexes involving first-row d-block metals are frequently reported in the literature. This phenomenon has been attributed to their variable oxidation states, coordination numbers, and geometries, which are conducive to adjusting their biological activity.^14^ Of particular note are copper, zinc and iron, which not only play crucial roles in various fundamental biological processes and are frequently linked to the active sites of many proteins and enzymes^41,42^ but are also affordable and widely available. Their dual attributes as biologically relevant and economically accessible have rendered them desirable candidates for therapeutic applications,^14^ resulting in a significant body of research on their antifungal potential. Consequently, these biometals are particularly well-suited to the present article, offering a unique opportunity to investigate crucial aspects such as ligand–metal interactions, SARs and the mechanisms underlying their antifungal activities. In addition, gallium has attracted considerable attention in recent years due to its distinctive properties in antimicrobial therapy, specifically its low toxicity to human cells and broad-spectrum activity against multidrug-resistant strains.^43^ It is also noteworthy that gallium serves as a radiopharmaceutical agent in nuclear medicine, thus potentially transcending its traditional diagnostic applications.^44^ As a result, it is hypothesized that gallium-based compounds have the potential to become the next generation of antimicrobials.^45^ Therefore, the inclusion of gallium complexes in this article provides a more comprehensive and up-to-date perspective, considering that gallium-based treatment is expected to become a new antifungal approach, particularly in the context of rising resistance.

The following sections illustrate, in chronological order, the chemical structures, activities, and SAR of a selected number of organic molecules from diverse classes, encompassing Schiff bases, sulfonamides, thiosemicarbazones, pyridines, and carboxamides, along with their corresponding Cu­(II), Zn­(II), Fe­(III) and Ga­(III) complexes. Throughout this article, a comprehensive and critical approach has been adopted to analyze a number of important examples from a medicinal chemistry perspective, elucidating the full therapeutic potential of these compounds through detailed key structural insights wherever possible.

### 2.1 Copper-Based Compounds

Copper-based compounds have attracted considerable attention in antimicrobial therapy due to their diverse mechanisms of action and applications.^46,47^ In particular, the described synergy between copper and antifungal azoles has fuelled this interest. For instance, studies on Cu­(II)-fluconazole complexes have shown that fluconazole (**FLZ**), the most commonly prescribed antifungal agent, is more effective against *Candida* spp. when coordinated with copper.^48^ Zabek et al.^49^ showed that [Cu_2_(**FLZ**)_2_(H_2_O)_2_]^2+^ exhibited fungicidal activity against **FLZ**-resistant strains of *C. glabrata*, just as Stevanović et al.^50^ observed that {[CuCl_2_(**FLZ**)_2_]·5H_2_O}_*n*_ prevented hyphae formation of *C. albicans*, affected biofilm formation of both *C. albicans* and *Candida parapsilosis*, and reduced the total amount of ergosterol in *C. albicans*; it was also found to be cytotoxic to human fibroblast MRC-5 cells, but to be more selective for *Candida* spp.

In most reported studies of synthetic Cu­(II)-based compounds, both the ligand(s) and the copper complexes are evaluated, with the complexes often showing superior performance. These copper complexes not only outperform the corresponding ligand(s), but also exhibit efficacy against a broad spectrum of fungal pathogens, thus rendering them a valuable tool in overcoming resistance.^51^ This section examines a number of ligand classes that demonstrate that coordination with Cu­(II) can enhance activity and provides insight into SARs. Moreover, the antifungal activities of some ligands in the presence of other metal ions and their antifungal activities are discussed in the following sections.

The investigation of 4-hydroxy-2*H*-pyrano­[3,2-*c*]­quinoline-2,5­(6*H*)-dione (**1**, Figure 1) and its copper complex [Cu­(L**1**)_2_] revealed their potential for inhibiting the growth of *C. albicans* and *Aspergillus flavus*.^52^ It is noteworthy that the study demonstrated that chelation to Cu­(II) significantly enhanced the efficacy of compound **1**. The free ligand showed only weak activity, with an average minimum inhibitory concentration (MIC) of 232.88 μM against *C. albicans* and 225.63 μM against *A. flavus*. In contrast, [Cu­(L**1**)_2_] showed moderate activity, with lower MIC averages of 53.96 and 47.94 μM, respectively.

**Figure 1:**
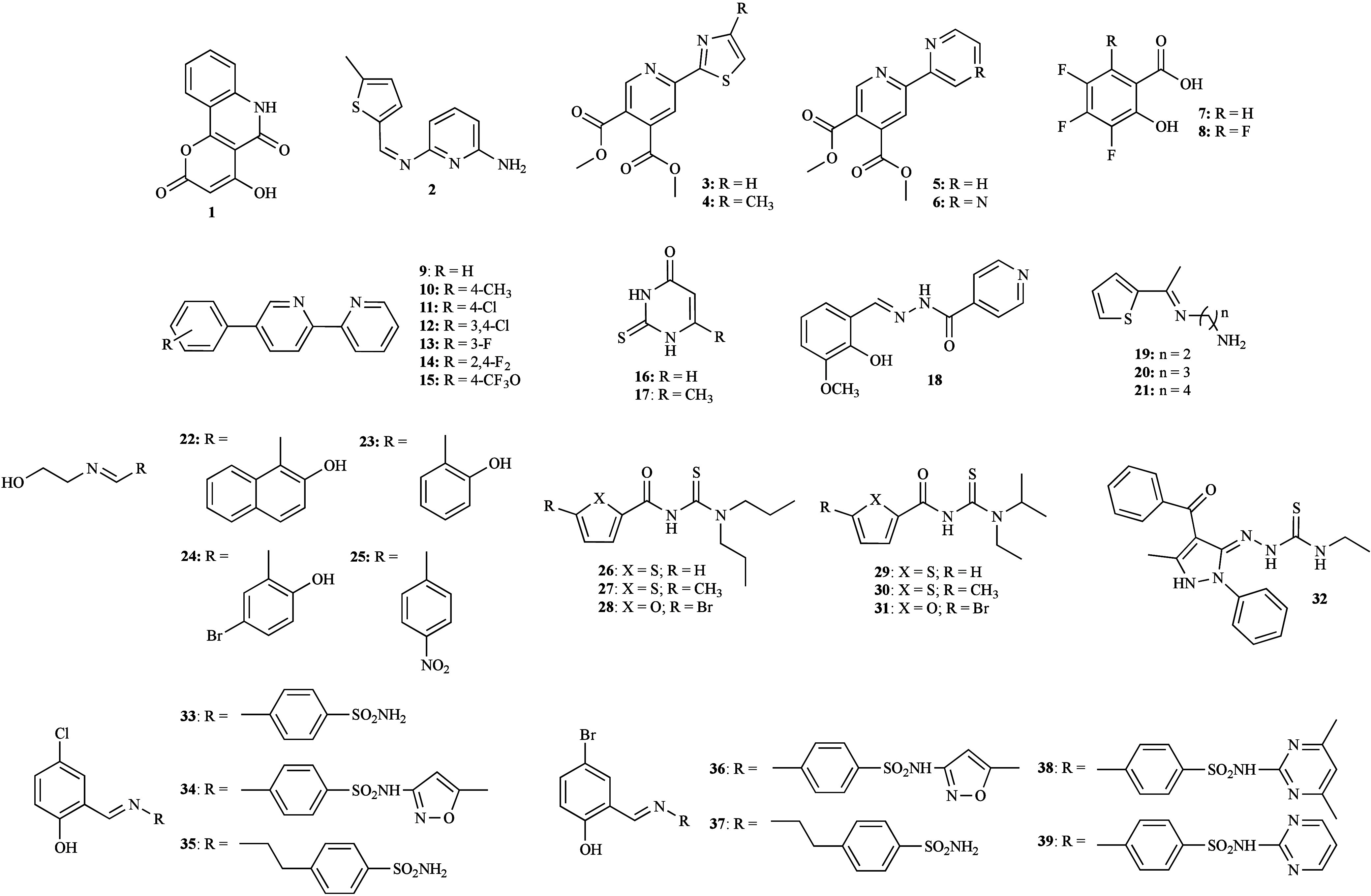
Structure of metal-free ligands **1**–**39**.

Further study involved a 5-methylthiophene Schiff base derivative (**2**, Figure 1) and its mixed copper complex [Cu­(L**2**)­(H_2_O)­Cl_2_]·4H_2_O, which were evaluated against the aforementioned fungal strains.^53^ While compound **2** was found to be inactive, [Cu­(L**2**)­(H_2_O)­Cl_2_]·4H_2_O showed remarkable activity, as evidenced by inhibition zone diameters (Ø inhibition) of 14 mm against *C. albicans* and 25 mm against *A. flavus*. This finding highlights the significant enhancement in efficacy observed upon coordination with Cu­(II).

Another study explored the role of a series of pyridine-4,5-dicarboxylate derivatives (**3**–**6**, Figure 1) and their corresponding heteroligand copper complexes (Figure 2) against *C. albicans* ATCC 10231 and *C. parapsilosis* ATCC 22019. It was observed that none of the metal-free ligands affected fungal growth,^54^ in contrast to their respective complexes.^55,56^ The coordination of these complexes to the Cu­(II) in the presence of dimethyl sulfoxide (DMSO), a common solvent for preparing stock solutions for biological screening, was monitored by UV–Vis spectroscopy. The results indicated that all complexes were stable in solution for up to 48 h, as evidenced by the absence of significant changes in the intensity and position of the absorption immediately after dissolution and at 24 and 48 h. Coordination with Cu­(II) generally enhanced anti-*Candida* activity, with two exceptions; [Cu­(L**4**–**5**)­(NO_3_)_2_(H_2_O)] against *C. parapsilosis* and [Cu­(L**6**)_2_(CF_3_SO_3_)­(H_2_O)]­CF_3_SO_3_·2H_2_O against *C. albicans*. Of the complexes tested, those based on compound **4** were identified as the most potent. In particular, [Cu­(L**4**)_2_Cl]_*n*_ exhibited the highest activity (MIC 31.25 and 250 μg/mL, respectively) while being inactive against bacteria and relatively non-toxic to healthy human fibroblast MRC-5 cells (drug concentration required for 50% inhibition, IC_50_ 70 mg/mL), indicating a favorable safety profile. Furthermore, the study revealed that all of the tested complexes, except [Cu­(L**4**–**5**)­(NO_3_)_2_(H_2_O)], exhibited near-complete inhibition of hyphal formation of *C. albicans* and that [Cu­(L**3**)­(NO_3_)­(H_2_O)_3_]­NO_3_ and [Cu­(L**3**)_2_Cl_2_] were effective inhibitors of the biofilm formation of *C. albicans*. It is well established that hyphal and biofilm formation contribute significantly to resistance against several antifungal agents. Currently, only a limited number of azoles (e.g., miconazole, **MCZ**), polyenes (e.g., liposomal formulations of amphotericin B), and echinocandins have shown efficacy against fungal biofilms.^57^ Consequently, these properties may prove the value of metal complexes for the management of drug-resistant infections, particularly in cases of invasive candidiasis, either as a monotherapy or in combination with antifungals commonly used in clinical practice but lacking antibiofilm efficacy against *C. albicans*, such as **FLZ**.^58^

**Figure 2:**
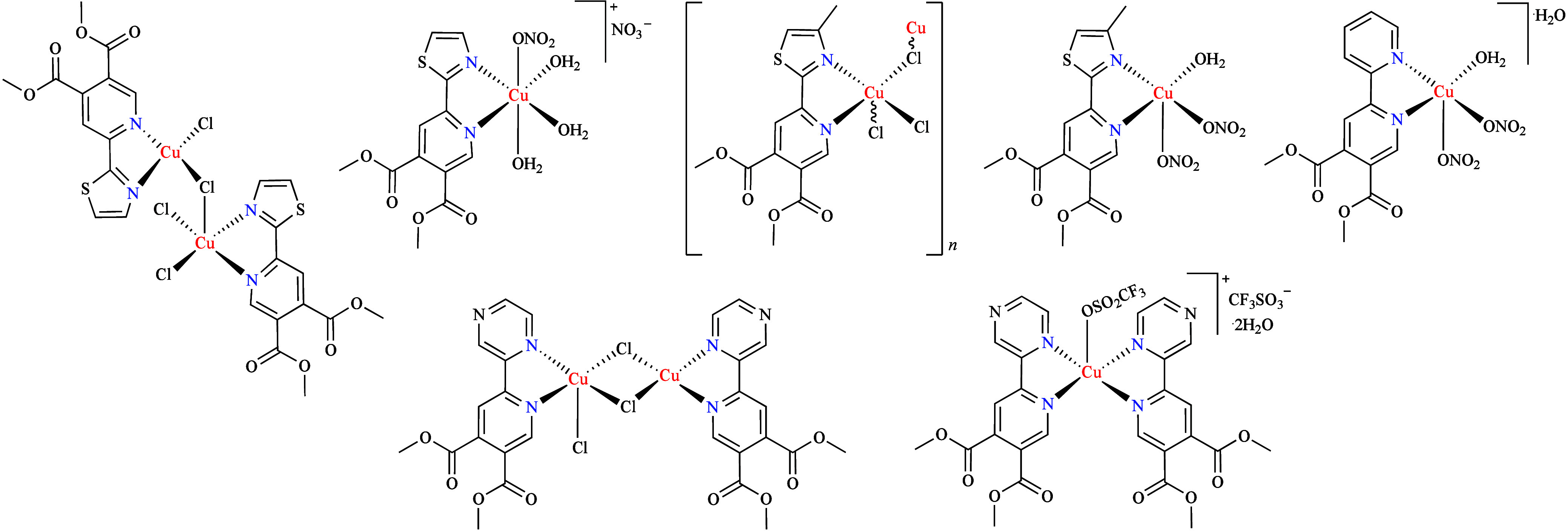
Schematic presentation of the copper complexes derived from compounds **3**–**6**.

The antifungal activity of polyfluorosalicylic acids (**7**–**8**, Figure 1) and their copper complexes was investigated against a variety of fungal strains, including *C. albicans*, *Epidermophyton floccosum*, and *Microsporum canis*.^59^ Ligand **7**, in contrast to compound **8**, was not active; the presence of an additional fluorine atom (electron-withdrawing group, EWG) contributed to a moderate activity for *Trichophyton rubrum*, *Nannizia gypsea* (formerly *Microsporum gypseum*), and *Trichophyton tonsurans* (MIC 25 μg/mL) and a weak activity against *Trichophyton violaceum* (MIC 50 μg/mL). The tested complexes showed negligible activity, with no significant disparities observed between them, except for [Cu­(L**7**)*py*H_2_O] and [Cu­(L**8**)*phen*]. Cu­(II) chelate of **7** with 2,2′-bipyridine was the only one to reveal an anti-*C. albicans* activity (MIC 50 μg/mL), while Cu­(II) chelate of **8** with 1,10-phenanthroline was the most active against four dermatophyte strains, exhibiting moderate-to-significant activity (MIC 6.25–25 μg/mL for *T. rubrum*, *N. gypsea*, *T. tonsurans*, and *Trichophyton interdigitale*).

A subsequent study by the same group involved a series of 5-aryl-2,2′-bipyridines (**9**–**15**, Figure 1) and their respective heteroligand complexes with salicylic acid (*sal*) and the aforementioned polyfluorosalicylic acids **7**–**8** to define their activity against the same fungal spectrum and an additional dermatophyte strain, *Trichophyton schoenleinii*.^60^ Compound **10** was identified as the most active among the free ligands, showing even better activity (MIC ≤ 12.7 μM) than **FLZ** (MIC ≤ 20.4 μM) against a greater number of clinical dermatophyte strains. Furthermore, the unsubstituted analogue **9** exhibited a slightly higher MIC against the same strains (MIC 26.9 μM), demonstrating that the presence of a methyl group (electron-donating group, EDG) is more beneficial for the activity (Figure 3). The replacement for fluorine atoms (EWG) in the phenyl ring resulted in a significant decrease in the antifungal efficacy; compound **13** showed moderate-to-significant activity against *T. interdigitale* and *M. canis*, and weak activity against *T. tonsurans* and *E. floccosum* as difluoro-substituted **14**. In contrast, the presence of chlorine atoms (EWG - compounds **11** and **12**) or a trifluoromethoxy group (EWG -compound **15**) resulted in a loss of antifungal efficacy (Figure 3). Regarding Cu­(II) complexation, the biological activity was typically equivalent to or greater than that of the bipyridines except for [Cu­(L**9**–**10**)_2_(L**7**)]·2H_2_O; therefore, the series of trifluorosalicylate complexes was not further developed. It is important to note that the stability of Cu­(L**11**)_2_(L**7**)]·2H_2_O was monitored and confirmed by means of elemental analysis and infrared (IR) spectroscopy, including under the conditions of biological experiments. The Cu­(II) complexes with salicylic acid as a secondary ligand manifested variable activity against the tested fungi. Except for *T. rubrum* and *C. albicans*, complex [Cu­(L**10**)_2_(*sal*)] inhibited the growth of almost all strains at the lowest concentrations (MIC ≤ 13.0 μM), yet was overall slightly less effective than its ligand. Additionally, the activity of compound **9** had no significant effect on Cu­(II) coordination against the tested fungi. Nevertheless, an increase in the efficacy was observed for compounds **13** and **14** upon complexation in most of the pathogens. Interestingly, complexes [Cu­(**11**–**13**)_2_(*sal*)] based on the inactive ligands demonstrated weak to moderate activity in the majority of the cases. These results are consistent with the existing literature, which documents the antifungal properties of metal complexes of salicylic acid with azo-ligands^61^ and the anti-*C. albicans* activity of complexes of Cu­(II) salicylate with bipyridine.^62^ On the other hand, the set of Cu­(II) complexes from tetrafluorosalicylic acid showed a more favorable impact on the activity of the compounds. In general, all the complexes displayed significantly lower MIC values than the free ligands. Notably, [Cu­(L**9**–**10**)_2_(L**8**)_2_] were the most active and even presented a better effect than **FLZ** (except for *T. interdigitale* and *C. albicans*). For the compounds/complexes that showed anti-*C. albicans* activity, the fungal spectrum was further extended to other clinically relevant species of the genus *Candida*. In comparison with **FLZ**, the compounds **9**–**10** and complexes [Cu­(L**9**–**10**)_2_(L**8**)_2_] were more effective against almost all the tested strains. Notably, compound **9** inhibited the growth of *Candida krusei*, *C. glabrata*, *Candida tropicalis*, and *Candida dubliniensis* at concentrations 6–12 times lower than those required for **FLZ**. This finding serves to further emphasize the potential of metal complexes as a promising alternative to traditional antifungal drugs.

**Figure 3:**
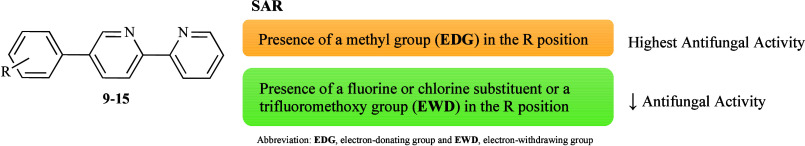
Putative SAR for the antifungal activity of ligands **9**–**15**.

The *in vitro* susceptibility of various clinical isolates of *Candida* to two chloro-copper complexes from 2-thiouracil derivatives (**16**–**17**, Figure 1) was determined.^63^ Initially, the stability of the complexes in solution (H_2_O/DMSO) was determined through UV–Vis spectroscopy 36 h after their preparation. The identical spectral behavior observed after 36 h indicates that the two complexes maintained their structures in solution and that no disruption of the metal–ligand bonds occurred. Among the tested complexes, [Cu­(L**16**)*bipy*Cl_2_] displayed fungicidal activity against several *Candida* species, with *C. krusei* being the most sensitive (MIC 31.25 μg/mL). In contrast, both the tested compounds and [Cu­(L**17**)*bipy*Cl_2_] were inactive, suggesting that the methyl substituent (EDG) on the thiopyrimidone ring could be considered unfavorable for enhancing the efficacy of compound **17** by Cu­(II) complexation (Figure 4). Interestingly, the noteworthy antifungal activity of [Cu­(L**16**)*bipy*Cl_2_] may be related to the synergistic effect between 2-thiouracil and the copper ion, as indicated by the MIC values of 1000 μg/mL observed for CuCl_2_ alone in all isolates tested. Furthermore, [Cu­(L**16**)*bipy*Cl_2_] significantly reduced the biofilm formation and preformed biofilms of certain *Candida* species, including *C. krusei* and *C. glabrata*. This was evidenced by a diminish in the number of viable cells within the biofilms at a concentration approximately 16-fold the MIC.

**Figure 4:**
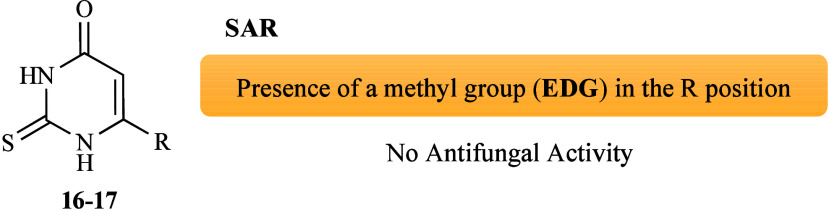
Putative SAR for the antifungal activity of copper complexes derived from ligands **16** and **17**.

The antifungal activity of four copper complexes based on *N*-isonicotinoyl-*N′*-(3-methoxy-2-hydroxybenzaldehyde)-hydrazone (**18**, Figure 1) was investigated against *C. albicans*.^64^ Apart from [Cu­(L**18**)­CH_3_COOH], all complexes exhibited potent fungicidal activity against the tested yeast, higher than that observed for nystatin. The most active complex was found to be [Cu­(L**18**)­ClO_4_]·H_2_O, with a MIC of 0.7 μg/mL, followed by [Cu­(L**18**)­NO_3_]·H_2_O and [Cu­(L**18**)­Cl]·2H_2_O (MIC 1.5 and 7.0 μg/mL, respectively). This suggests that the presence of the perchlorate ion within the coordination sphere is critical for efficacy. Regrettably, the precise role of Cu­(II) chelation in enhancing activity remains unclear, as the efficacy of the metal-free ligand **18** was not investigated. In the same study, the effect of the complexes on the proliferation of breast cancer cell lines (MCF-7, SKBR-3, A375, and NCI-H1573) was investigated. The complexes [Cu­(L**18**)­CH_3_COOH], [Cu­(L**18**)­ClO_4_]·H_2_O, and [Cu­(L**18**)­NO_3_]·H_2_O showed a notable improvement of the cytotoxic effect in all four cell lines tested, compared to the minimal cytotoxicity of their ligand. In contrast, [Cu­(L**18**)­Cl]·2H_2_O showed a variable response depending on the cell type, demonstrating increased cytotoxicity on MCF-7 and SKBR-3.

A series of thiophene-derived Schiff bases (**19**–**21**, Figure 1) and their copper complexes [Cu­(L**19**–**21**)_2_]­Cl_2_ were evaluated for their potential as inhibitors against *C. albicans*, *C. glabrata*, *A. flavus*, *Fusarium solani*, *M. canis*, and *Trichophyton longifusus*.^65^ Like its ligands, [Cu­(L**19**)_2_]­Cl_2_ was consistently more active, followed by [Cu­(L**20**)_2_]­Cl_2_ and [Cu­(L**21**)_2_]­Cl_2_, implying that the length of the spacer between the amines can influence the antifungal efficacy (Figure 5). The complexation with Cu­(II) resulted in a noteworthy improvement effect (58% average inhibition) in comparison to the uncomplexed Schiff′s bases (37% average inhibition).

**Figure 5:**
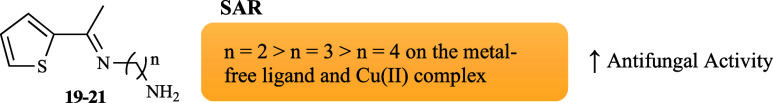
Putative SAR for the antifungal activity of ligands **19**–**21** and their copper complexes.

The role of a library of ethanolamine-derived compounds (**22**–**25**, Figure 1) and respective copper complexes [Cu­(L**22**–**25**)_2_] on the growth of the same representative strains mentioned above was investigated.^66^ Among the free ligands, compounds **24** and **25** were the most effective, displaying similar moderate activities in all cases (59 and 60% average inhibition, respectively). This indicates that the presence of an *ortho*-nitro substituent attached to an aromatic ring appears to be important and that the additional introduction of an *ortho*-bromo group to a phenol may hold greater potential (Figure 6). The complexes showed increased activity (62% average inhibition) rather than their non-coordinated counterparts (47% average inhibition) of which Cu­(L**23**)_2_] showed a particularly marked enhancement. It can be argued that the bromo and nitro substituents (EWG) are potentially accountable for the higher effectiveness of the complexes (Figure 6), as their ligands, being [Cu­(L**24**)_2_] more active (69% average inhibition) than [Cu­(L**25**)_2_] (66% average inhibition). These findings exposed the considerable impact of strategic substitution and metal coordination in optimizing the antifungal properties of these compounds.

**Figure 6:**
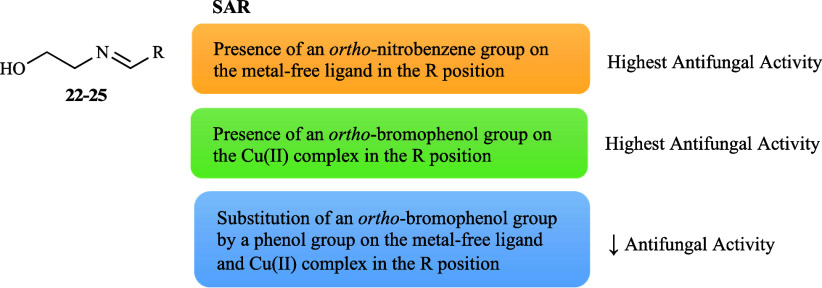
Putative SAR for the antifungal activity of ligands **22**–**25** and their copper complexes.

Another research group studied the effect of six thiourea (carboxamide) derivatives (**26**–**31**, Figure 1) and their copper complexes [Cu­(L**26**–**31**)_2_] on the aforementioned fungal strains.^67,68^ Although the ligands were poorly active against most of the tested strains, compounds **26**–**31** showed moderate activity against a representative fungus; **26** and **29** against *C. albicans* (50 and 53% inhibition, respectively), **27** against *M. canis* (63% inhibition), and **28**, **30**–**31** against *A. flavus* (52, 57 and 50% inhibition, respectively). Compound **30** also moderately affected *C. glabrata* (62% inhibition). Among the free ligands, compounds **28** and **29** displayed the highest activities (42 and 43% average inhibition, respectively), concluding that the presence of a 5-bromofuran seems to be more favorable for the antifungal effect and that an *N*-ethylpropan-2-amine is more advantageous than a dipropylamine (Figure 7). The Cu­(II) complexes were found to be more active (44% average inhibition) than the metal-free ligand (40% average inhibition), underscoring the advantageous impact of Cu­(II) coordination. The enhancement of the inhibitory effect of compounds **29**–**31** upon chelation was more pronounced compared to compounds **26**–**28**, showing the potential for structural modifications to increase activity, paving the way for more potent therapeutic candidates. It was observed that all complexes demonstrated moderate activity on the same strains as their ligands, except [Cu­(L**28**)_2_], which, in a manner analogous to [Cu­(L**31**)_2_], also exhibited an effect against *C. glabrata*.

**Figure 7:**
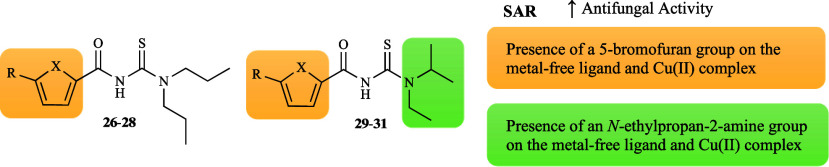
Putative SAR for the antifungal activity of ligands **26**–**31** and their copper complexes.

The impact of a mixed copper complex derived from 1-phenyl-3-methyl-4-benzoyl-5-pyrazolone-4-ethyl-thiosemicarbazone (**32**, Figure 1) against *C. albicans* ATCC 10231 was investigated.^69^ The antifungal data indicated that ligand **32** was inactive, in contrast to its metal complexes; [Cu­(L**32**)­Cl]·C_2_H_5_OH, [Cu­(L**32**)_2_]·H_2_O, [Cu­(L**32**)­Br]·H_2_O, and [Cu­(L**32**)­NO_3_]·2C_2_H_5_OH displayed potent fungicidal effect with MIC values of 1.4–1.5 μg/mL. Indeed, the anti-*C. albicans* activity of ligand **32** was enhanced by 57 to 53-fold in comparison to nystatin, thus demonstrating the beneficial impact of Cu­(II) chelation and its potential as a highly effective antifungal strategy. The potential of the ligand **32** and its complexes to inhibit the proliferation of human leukemia HL-60 cells was assessed. The ligand demonstrated no inhibitory activity when tested at all three concentrations. However, the copper complexes revealed notable antiproliferative effects. The impact of halogens in the coordination sphere ([Cu­(L**32**)­Br]·H_2_O and [Cu­(L**32**)­Cl]·C_2_H_5_OH) was found to be less pronounced, whereas the copper capsulated with two ligands ([Cu­(L**32**)_2_]·H_2_O) exhibited the highest activity for HL-60 cells These results underline the pivotal role of the metal ion in the Schiff bases composition.

A series of sulfonamide-derived Schiff bases (**33**–**39**, Figure 1) and their copper complexes [Cu­(L**33**–**39**)_2_(H_2_O)_2_] were screened against *C. albicans*, *C. glabrata*, *A. flavus*, *F. solani*, *M. canis*, and *T. longifucus*. Among the 5-chloro (**33**–**35**)^70^ and 5-bromo ligands (**36**–**39**),^71,72^ compounds **33** and **39** were found to be the most active (47 and 53% of the average inhibition, respectively). This indicates that the presence of a methylene group between the Schiff base and benzenesulfonamide plays a more important role, as well as the presence of an unsubstituted pyrimidine. Furthermore, the nature of the halogen group at position C-5 can influence the inhibitory effect, with a chloro substituent (EWG) appearing to be more favorable when compared to a bromo substituent (EWG) (Figure 8). Complexation with Cu­(II) resulted in a higher biological activity than the corresponding ligands, except for [Cu­(L**34**)_2_(H_2_O)_2_] and [Cu­(L**39**)_2_(H_2_O)_2_]. Interestingly, a significant increase was observed for [Cu­(L**33**)_2_(H_2_O)_2_], [Cu­(L**35**)_2_(H_2_O)_2_], and [Cu­(L**38**)_2_(H_2_O)_2_]. The results showed a substantial discrepancy between the tested complexes and their ligands. Particularly, the series of 5-bromo ligands showed that [Cu­(L**38**)_2_(H_2_O)_2_] displayed superior activity (50% average inhibition), demonstrating that the introduction of a dimethyl-substituent (EDG) attached to the pyrimidine was responsible for the enhanced effectiveness of the complex, and that compound **36** was more active than compound **34** (Figure 8).

**Figure 8:**
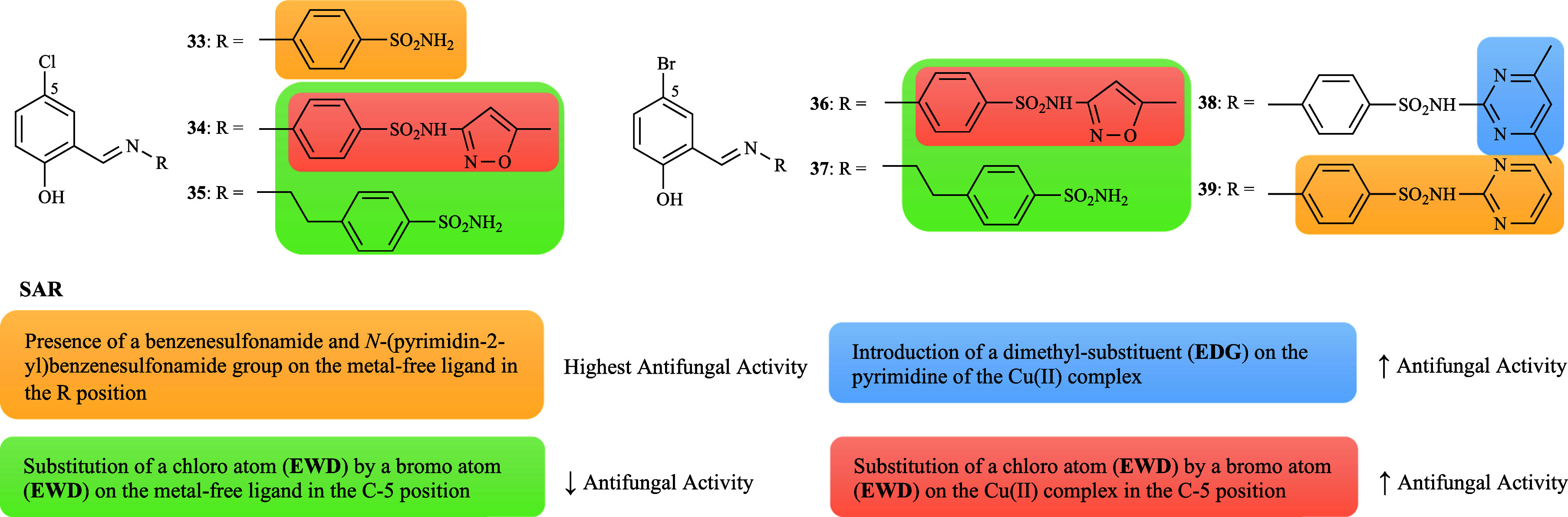
Putative SAR for the antifungal activity of ligands **33**–**39** and their copper complexes.

### 2.2 Zinc-Based Compounds

Zinc coordination can potentiate the antifungal activity while providing a new avenue for targeting difficult-to-treat infections.^73^ As a result, classical azole agents have proven to be excellent ligands for the development of zinc-based antifungal compounds with improved efficacy and therapeutic potential. For example, Azevedo-França et al.^33^ showed that zinc complexes derived from ketoconazole (**KTZ**) and **CTZ** exhibit broad-spectrum activity against pathogens such as *C. albicans* and *Cryptococcus neoformans* at lower concentrations compared to their parent drugs and even to **FLZ**, a commonly prescribed drug for these infections. Specifically, complexes such as [Zn­(**KTZ**)_2_(CH_3_COO)_2_]·2H_2_O and [Zn­(**KTZ**)_2_(NO_3_)­(H_2_O)]­NO_3_ were found to induce significant morphological changes in *Sporothrix brasiliensis*, including yeast-to-hyphae conversion, increased cell size, and cell wall damage. It is noteworthy that these zinc complexes demonstrated higher selectivity for the three species over mammalian cells, although they exhibited greater cytotoxicity compared to **KTZ** in assays with LCC-MK2 cells.

Although well documented, the amount of research on zinc-based compounds is comparatively less extensive than that on copper-based compounds. The preponderance of extant data indicates that zinc complexes exhibit enhanced activity in comparison to the ligand alone, where both the ligand and its corresponding complex are evaluated. Most of the ligands evaluated with Zn­(II) have also been assessed with Cu­(II) and it is typically observed that zinc complexes are as active or more active than their copper analogues. This section presents a selection of zinc-based antifungal compounds reported in the literature, highlighting the positive effect of coordination with Zn­(II) on activity. Additionally, insights into SARs are elucidated, along with a comparative analysis of zinc-based versus copper-based antifungal compounds in some studies.

In addition to the copper complexes, zinc complexes of the set of thiophene-derived Schiff bases (**19**–**21**, Figure 1) were also tested.^65^ Similar to the copper complexes, the activity of the ligands increased significantly when coordinated with Zn­(II) (59% average inhibition), with [Zn­(L**21**)_2_]­Cl_2_ showing a higher improvement effect. [Zn­(L**19**)_2_]­Cl_2_ displayed the most pronounced percentage of growth inhibition among the zinc complexes (Figure 9) and even the metal complexes. It is also noteworthy that [Zn­(L**19**–**20**)_2_]­Cl_2_ were more active when compared to their copper analogues, which serves to illustrate the critical role of the nature of the metal ions in modulating the activity of these Schiff bases.

**Figure 9:**
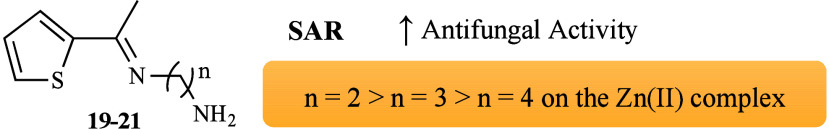
Putative SAR for the antifungal activity of zinc complexes derived from ligands **19**–**21**.

The zinc complexes of the ethanolamine-derived compounds (**22**–**25**, Figure 1) were also developed.^66^ Overall, a significant increase in activity was reported upon Zn­(II) complexation (63% average inhibition) against the tested strains in comparison to the free ligands. Among the metal complexes, the Zn­(II) complexes were found to be the most active, except for [Zn­(L**23**)_2_]. The high activity of [Zn­(L**25**)_2_] followed by [Zn­(L**24**)_2_] showed the importance of nitro and bromo substituents (EWG), similar to those observed for their ligands and copper analogues; nevertheless, an *ortho*-nitro substituent was more promising than the *ortho*-bromo substituent (Figure 10), suggesting a distinctive advantage for this functional group in the zinc complexes. This is in contrast to what was observed for the copper complexes and points to the nuanced role of metal coordination in optimizing antifungal efficacy.

**Figure 10:**
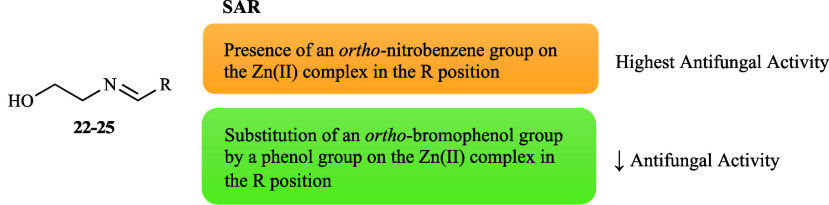
Putative SAR for the antifungal activity of zinc complexes derived from ligands **22**–**25**.

The antifungal properties of zinc complexes with the six carboxamide derivatives (**26**–**31**, Figure 1) were additionally explored.^67,68^ Overall, [Zn­(**26**–**30**)_2_] followed the trend observed for the copper complexes, in which the coordination to Zn­(II) significantly improved the activity of the metal-free ligands (43% average inhibition). However, the activity of the corresponding zinc complexes was slightly inferior to that of copper (44% average inhibition), in contrast to the complexes [Zn­(L**28**)_2_] and [Zn­(L**30**)_2_]. In addition, the antifungal data showed that [Zn­(L**28**)_2_] and [Zn­(L**31**)_2_] were the most active (44 and 45% average inhibition, respectively) compared to the zinc complexes with thiophene ring (41–43% average inhibition) (Figure 11).

**Figure 11:**
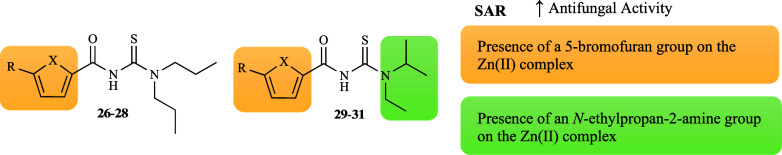
Putative SAR for the antifungal activity of zinc complexes derived from ligands **26**–**31**.

The zinc complexes based on the series of sulfonamide-derived Schiff bases (**33**–**39**, Figure 1) were also investigated.^70–72^ As with the copper complexes, the ligands’ efficacy increased significantly upon coordination with Zn­(II), in contrast to compounds **33**–**34**. With some exceptions, comparable activities were observed between [Zn­(L**36**)_2_(H_2_O)_2_] and [Zn­(L**38**)_2_(H_2_O)_2_] (56.3 and 56% average inhibition, respectively), and [Zn­(L**37**)_2_(H_2_O)_2_] and [Zn­(L**39**)_2_(H_2_O)_2_] (58% average inhibition). [Zn­(L**36**–**39**)_2_(H_2_O)_2_] exhibited superior activity, which can be explained by the presence of a bromo-substituent (EWG), a pattern also observed in the analogues copper complexes (Figure 12). Furthermore, the methylene group between the Schiff base and benzenesulfonamide can no longer be considered essential for activity (Figure 12) as was initially assumed. To illustrate, [Zn­(L**34**)_2_(H_2_O)_2_] showed a higher inhibitory effect when compared to [Zn­(L**33**)_2_(H_2_O)_2_], a finding that contrasts with the results observed for copper complexes. In conclusion, the Zn­(II) complexes of ligands **36**–**39** were slightly more active than the respective Cu­(II) complexes.

**Figure 12:**
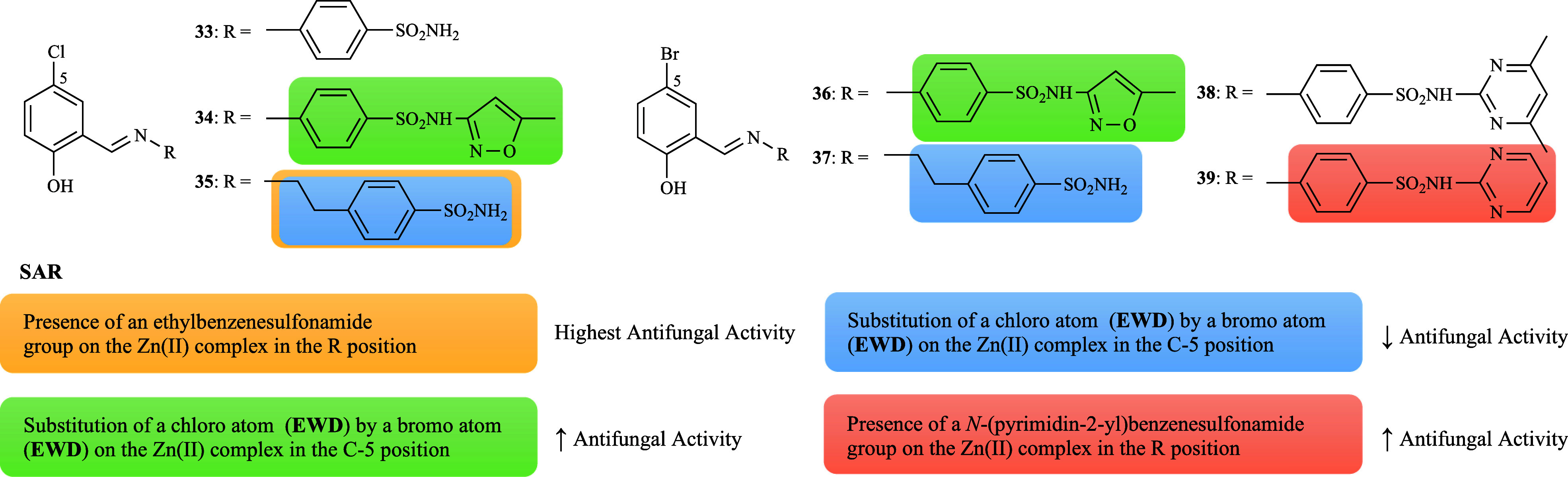
Putative SAR for the antifungal activity of zinc complexes derived from ligands **33**–**39**.

Three nitrogen-based heterocycles (**40**–**42**, Figure 13) and their chloro-zinc complexes [Zn­(L**40**)_2_Cl_2_], [Zn­(L**41**)­Cl_2_]_*n*_, and [Zn­(L**43**)_2_Cl_2_] were prepared^74^ and a preliminary evaluation of the antifungal activity was conducted using the disk-diffusion assay against *C. albicans* ATCC 10231 and *C. parapsilosis* ATCC 22019. Compounds **40** and **41** were inactive, in contrast to compound **42**, complexes and ZnCl_2_ which induced observable zones of inhibition. The stability of the zinc complexes in DMSO and deuterated DMSO (DMSO-*d*_6_) was monitored by UV–Vis and Nuclear Magnetic Resonance (NMR) spectroscopy, after dissolution and after 48 h, respectively. The NMR spectra indicated that the *N*-heterocycle remained coordinated to the Zn­(II) for 48 h, with no evidence of DMSO coordination. In the UV–Vis spectra, a slight decrease in the intensity of the absorption maxima was observed after 48 h compared to the initial state immediately after dissolution. However, the shape and position of the absorption maxima remained unaltered. The spectroscopic data collectively substantiate the sufficient stability of the complexes in a DMSO solution. Subsequent microdilution susceptibility testing of *Candida* spp. revealed that compounds **40** and **41** displayed inactivity toward the *Candida* species, as their zinc complexes. Conversely, [Zn­(L**42**)_2_Cl_2_] and its ligand were weakly effective (MIC 125–250 and 200–250 μg/mL, respectively), along with ZnCl_2_ (MIC 250 μg/mL), against several *Candida* species. Interestingly, the coordination of compound **42** with Zn­(II) resulted in either equivalent or reduced biological activity, except for *C. krusei*; [Zn­(L**42**)_2_Cl_2_] inhibited the growth of *C. krusei* at a concentration that was 2-fold lower than that observed for the corresponding ligand. This finding is intriguing as it challenges the typical expectation that metal complexation enhances antifungal activity; however, the exception with *C. krusei* suggests the existence of a specific interaction or mechanism that is particularly effective against this strain. Furthermore, cytotoxic studies showed that [Zn­(L**42**)_2_Cl_2_] was less toxic to human fibroblast MRC-5 cells (IC_50_ 60 mg/mL) when compared to [Zn­(L**40**)_2_Cl_2_] and [Zn­(L**41**)­Cl_2_]_*n*_ (IC_50_ 35 and 40 mg/mL, respectively), indicating a potentially safer profile. In this study, [Zn­(L**42**)_2_Cl_2_] also exhibited complete inhibition of hyphal formation, efficiently prevented *C. albicans* adhesion to the layer of A549 lung carcinoma cells and showed an impressive synergistic effect with nystatin at subinhibitory concentrations. Despite its weak activity, the aforementioned attributes suggest that [Zn­(L**42**)_2_Cl_2_] could prove a valuable addition to combination therapies, especially for the treatment of biofilm-related infections as the ability to change morphologically is known to be important in the development of fungal biofilms. Moreover, the complex has shown promise in the prevention of *C. albicans* colonization.^75^

**Figure 13:**
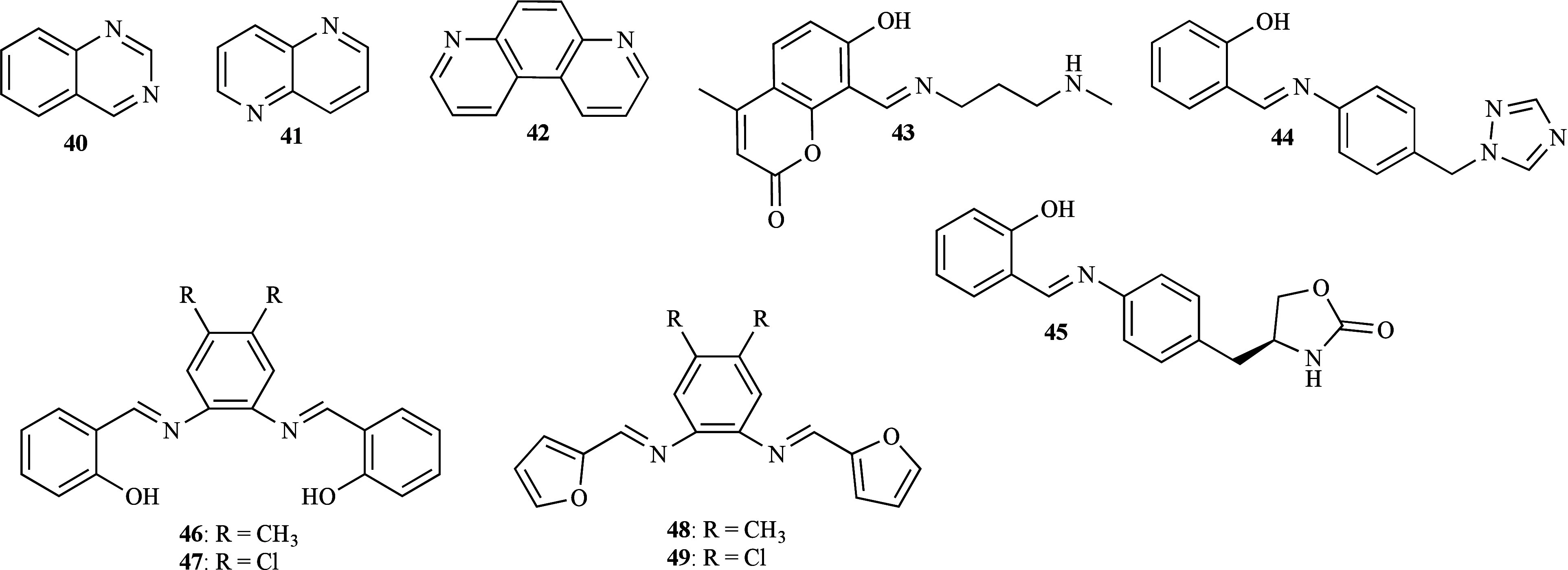
Structure of metal-free ligands **40**–**49**.

In a separate study, the effect of three novel Schiff base derivatives (**43**–**45**, Figure 13) and respective zinc complexes [Zn­(L**43**–**45**)_2_]·2H_2_O against *C. albicans* ATCC 2091 and *Aspergillus niger* ATCC 9029 was studied.^76^ All compounds exhibited significant activity against *A. niger* (MIC 0.8–6.25 μg/mL) and moderate-to-significant against *C. albicans* (MIC 12.5–50 μg/mL). Among them and compared to FLZ (MIC 8 and 16 μg/mL), compound **45** was the most active with MIC values of 6.25 and 12.5 μg/mL against *A. niger* and *C. albicans*, respectively. No significant difference was observed between the complexes; however, only [Zn­(L**43**)_2_]·2H_2_O and [Zn­(L**44**)_2_]·2H_2_O against *C. albicans*, and [Zn­(L**45**)_2_]·2H_2_O against *A. niger* demonstrated higher efficacy compared to their free ligands. In particular, [Zn­(L**43**)_2_]·2H_2_O and [Zn­(L**44**)_2_]·2H_2_O against *C. albicans* (MIC 3.12 μg/mL) and all three complexes against *A. niger* (MIC 0.8 μg/mL for [Zn­(L**43**)_2_]·2H_2_O and 3.12 μg/mL for [Zn­(L**44**–**45**)_2_]·2H_2_O) showed more promising activity when compared to FLZ. This highlights the potential of metal complexes as a viable alternative to traditional antifungal drugs. The most active compound, [Zn­(L**43**)_2_]·2H_2_O, suggests that the 2*H*-pyran-2-one moiety incorporated within the Schiff base structure seems to be crucial for the enhancement of activity.

Two salen derivatives (**46**–**47**, Figure 13) and their respective zinc complexes [Zn­(L**46**–**47**)]·H_2_O were evaluated for their inhibitory potential against *C. albicans*, *A. niger*, *A. flavus*, and *F. solani*.^77^ The inhibitory effect was higher for all tested strains when complexed with Zn­(II) (Ø inhibition 17–25 mm), compared to the corresponding ligands (Ø inhibition 11–18 mm). As with its ligand, [Zn­(L**47**)]·H_2_O was found to be most active concluding that the increase in activity was more pronounced in the presence of a dichloro-substituent (EWG) compared to a dimethyl substituent (EDG) (Figure 14). A subsequent study involving two compounds (**48**–**49**, Figure 13), analogues to those previously mentioned, and their zinc complexes [Zn­(L**48**–**49**)]­(CH_3_COOH)_2_·H_2_O were carried out against *C. albicans* and *A. flavus*.^78^ Similar activities were observed against the two tested strains and no effect upon the complexation with Zn­(II) was noted. Both ligands and complexes were moderately active against *C. albicans* (Ø inhibition 9–10 mm) and inactive against *A. flavus*; these results suggest high selectivity for *C. albicans*. As previously indicated, the electron-withdrawing zinc complex displayed slightly superior activity compared to its electron-donating counterpart, like its parent ligands (Figure 14). Furthermore, these four tested complexes were found to be stable by thermal studies using thermogravimetric analysis (TGA) and the zinc complexes and their respective ligands derived from ligand groups with a difuran ([Zn­(L**48**–**49**)]·H_2_O) were more active against *C. albicans* and *A. flavus* than the analogue zinc complexes with diphenol ([Zn­(L**46**–**47**)]­(CH_3_COOH)_2_·H_2_O) (Figure 14), underscoring the importance of structural modifications in antifungal activity.

**Figure 14:**
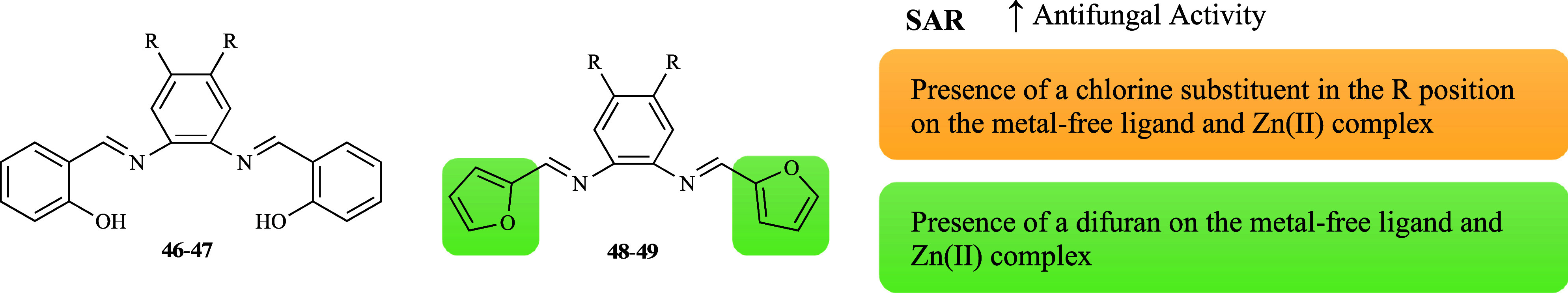
Putative SAR for the antifungal activity of ligands **46**–**49** and their zinc complexes.

### 2.3 Iron-Based Compounds

The literature on iron-based antifungal compounds is expanding, yet it remains comparatively limited in scope when contrasted with the extensive research on metal­(II)-based compounds, particularly those of Cu­(II) and Zn­(II). In recent years, there has been a notable increase in publications exploring the potential of iron in antifungal therapy, especially in the context of siderophores and their conjugation with antimicrobials to improve drug delivery and efficacy.^79^ One innovative example is the polymeric siderophore conjugate developed by Qian et al.,^80^ which combines poly­(l-lysine hydrochloride) (**PLL**), a natural broad-spectrum antimicrobial, with 2,3-dihydroxybenzoic acid (**DHBA**). This chelator showed a strong affinity for iron, mimicking the binding characteristics of natural siderophores such as enterobactin. Its mechanism of action involves the disruption of fungal iron acquisition through the siderophore iron assimilation pathway. The resulting **PLL-DHBA** conjugate exhibited potent antifungal activity, particularly against drug-resistant strains, and demonstrated superior efficacy to traditional antifungal agents such as voriconazole, posaconazole, and FLZ in specific species. In the context of this article, the focus should be on iron-based antifungals, particularly in light of the recent publication of a comprehensive review on siderophore-antifungal conjugates.^79^ The primary objective of conjugates is to improve drug delivery to fungal cells by exploiting iron acquisition mechanisms while in iron-based antifungals the metal coordination enables direct modulation of antifungal activity.

The development of iron-based azoles has attracted considerable interest, paving the way for the exploration of synthetic iron-based compounds, as has been the case with copper- and zinc-based compounds. For example, Abd El-Halim et al. demonstrated that iron complexes derived from clotrimazole (**CTZ**)^81^ and **MCZ** ^82^ exhibited significantly greater growth inhibition than their parent drugs against clinically relevant species like *C. albicans* and *A. fumigatus*. Given the lack of specific quantitative data, this section provides an overview of almost all iron-based antifungal compounds reported in the literature, where it has often been observed that the complexes outperform the metal-free ligands in inhibiting fungal growth when both are evaluated. Moreover, some of these ligands were also tested with Ga­(III), whose activities are described in the next section.

Along with the copper complex, an iron complex of 4-hydroxy-2*H*-pyrano­[3,2-*c*]­quinoline-2,5­(6*H*)-dione (**1**, Figure 1) was evaluated against *C. albicans* and *A. flavus*.^52^ The activity of compound **1** was found to be considerably enhanced when coordinated with Fe­(III), achieving an average MIC of 51.75 and 55.29 μM, respectively. Curiously, when compared with its copper analogue, [Fe­(L**1**)_2_Cl]·H_2_O demonstrated a more pronounced inhibitory effect toward *C. albicans* and a less pronounced effect toward *A. flavus*. These differential characteristics highlight a pivotal point regarding the impact of disparate metals on activity and that exploring diverse metal complexes could lead to more targeted and efficacious treatments.

The *in vitro* susceptibility of three fungal species 4-(2-hydroxyphenylazo)-1-naphthol (**50**, Figure 15) and its heteroligand iron complex [Fe­(L**50**)_2_Cl]·H_2_O were tested.^83^ The results indicated that compound **50** displayed a moderate antifungal effect, with *A. flavus* being the most susceptible (MIC 25 μg/mL) followed by *C. albicans* and *T. rubrum* (MIC 50 μg/mL). In contrast, the metal complex showed significantly higher activity with MIC values of 6.25 μg/mL against both *C. albicans* and *A. flavus,* and 12.5 μg/mL against *T. rubrum*. These findings suggest that coordination to Fe­(III) substantially increases the efficacy of the tested compound. The relative stability of the studied complex was supported by density functional theory (DFT) calculations.

**Figure 15:**
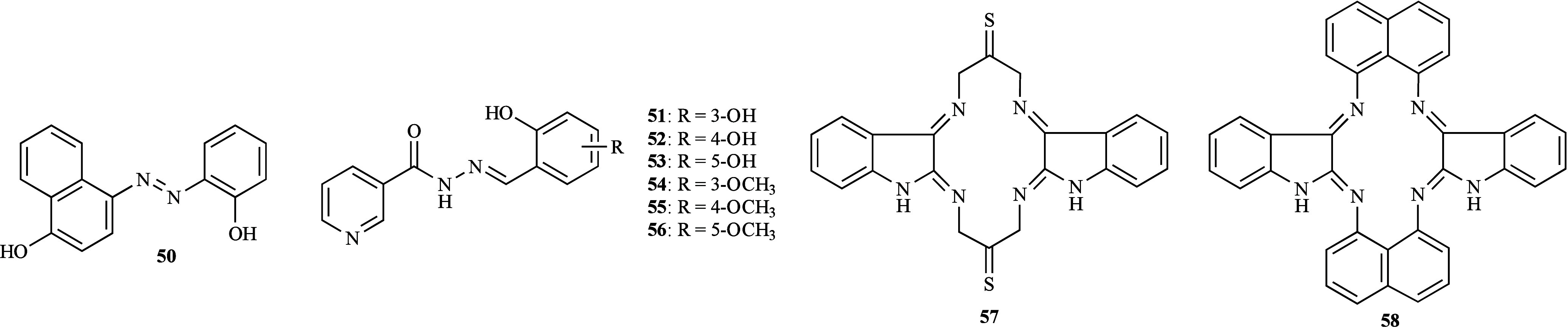
Structure of metal-free ligands **50**–**58**.

The antifungal activity of six aroylhydrazone derivatives (**51**–**56**, Figure 15) and their iron complexes [Fe­(L**51**–**56**)_2_] was evaluated against *C. albicans* ATCC 10213 and fluconazole-resistant *C. albicans* MFBF 11100.^84,85^ All the compounds were inactive toward both strains, except for compound **55**; the presence of a 4-methoxy substituent (EDG) contributed to a potent activity with a concentration value required for 90% inhibition (IC_90_) of 0.18 and 0.10 μM for ATCC 10213 and MFBF 11100, respectively. Interestingly, the tested compound did not present antibiofilm activity against the fluconazole-resistant strain at the tested concentrations. Nevertheless, antibacterial assays conducted within the same study showed that compound **55** selectively targeted fungi. For the iron complexes, two assays were performed against the same two representative *C. albicans* strains. Both the agar well-diffusion and microdilution broth methods showed that none of the complexes exhibited antifungal activity. Notably, [Fe­(L**55**)_2_] represents an atypical example where the activity of compound **55** decreases significantly upon Fe­(III) complexation.

Three iron macrocyclic complexes [Fe­(L**57**)­X]­X_2_ (X = Cl, NO_3_, and CH_3_COOH) were tested against *C. albicans* MTCC 227 and *Saccharomyces cerevisiae* MTCC 170.^86^ [Fe­(L**57**)­Cl]­Cl_2_ was the only complex to be effective showing a weak activity (Ø inhibition 11.3 and 11.5 mm, respectively). On the contrary, all the complexes were active against two *Aspergillus* species, but none of them restricted the fungal growth moderately to significantly. Among them, [Fe­(L**57**)­Cl]­Cl_2_ exhibited the highest percentage of mycelial growth inhibition against *A. niger* MTCC 282 and *A. flavus* MTCC 871 (44 and 39% inhibition, respectively). Since the antifungal activity of the metal-free ligand (**57**, Figure 15) was not reported, it is difficult to understand the effect of chelation with Fe­(III); nevertheless, it can be concluded that introducing chlorine (EWG) as a co-ligand resulted in more favorable antifungal activity. Another study conducted by the same group evaluated three additional iron macrocyclic complexes [Fe­(L**58**)­X]­X_2_ (X = Cl, NO_3_, and CH_3_COOH) for their inhibitory potential against the same fungal spectrum.^87^ [Fe­(L**58**)­Cl]­Cl_2_ was the most efficient to both *Aspergillus* species, presenting moderate activity (65 and 63% inhibition, respectively), followed by [Fe­(L**58**)­(NO_3_)]­(NO_3_)_2_ and [Fe­(L**58**)­(CH_3_COOH)]­(CH_3_COOH)_2_. These results reaffirmed that the chloro-iron complex is a more promising antifungal drug and that the presence of a naphthalene group led to a significant improvement in the antifungal activity when compared to [Fe­(L**57**)­Cl]­Cl_2_ (Figure 16).

**Figure 16:**
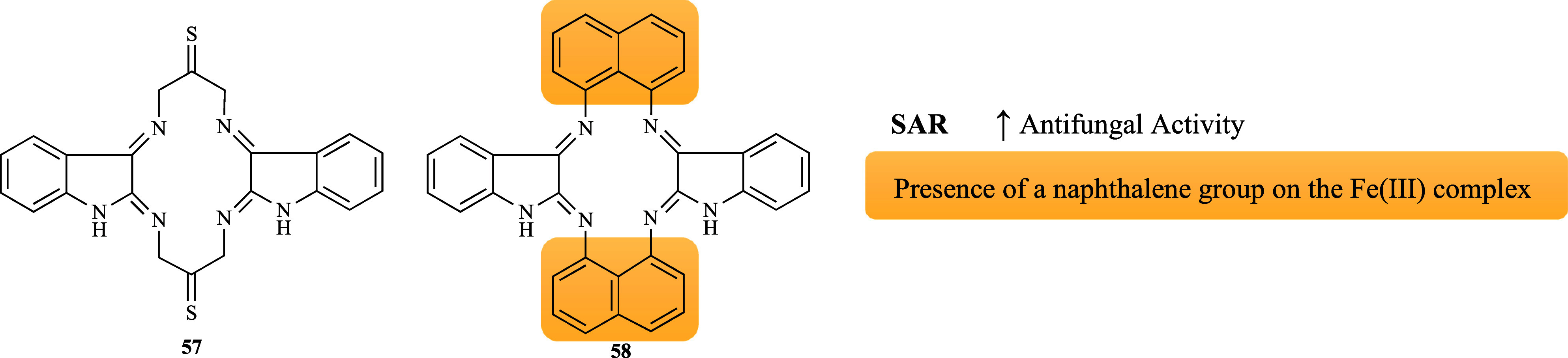
Putative SAR for the antifungal activity of iron complexes derived from ligands **57** and **58**.

### 2.4 Gallium-Based Compounds

Gallium-based compounds exhibit significant potential and distinct advantages as antifungal agents; their mechanism of action exploits the chemical similarity of gallium to iron, allowing them to disrupt iron-dependent fungal metabolic pathways.^45^ Furthermore, the “Trojan Horse” strategy which employs siderophores—molecules that can transport gallium into fungal cells by mimicking iron—provides a foundation for innovative drugs that enhance target therapeutic efficacy while also offering the potential for combination PET/CT (positron emission tomography/computed tomography) imaging (gallium-labeled).^88^ However, the amount of research conducted in this area is still insufficient in comparison to the extensive work done on metal-based antifungal compounds. Nevertheless, it is crucial to consolidate the research discoveries of gallium complexes in the literature. For that reason, this section summarizes the antifungal activities of all metal-free ligands and their gallium complexes reported in the literature, along with insights into SARs.

The gallium complexes of the six above-mentioned aroylhydrazone derivatives (**51**–**56**, Figure 15) were also tested against *C. albicans* ATCC 10213 and fluconazole-resistant *C. albicans* MFBF 11100 with the same two assays.^85^ From the diffusion method, it was observed that only the complexes of compounds **55**–**56** were active; [Ga­(L**55**)_2_] showed Ø inhibitions of 12.7 ± 1.2 and 12.3 ± 0.6 mm, respectively, and [Ga­(L**55**)_2_] produced a zone of inhibition of 10.7 ± 0.6 mm against *C. albicans* ATCC 10213. However, the microdilution method yielded divergent results. Apart from [Ga­(L**55**–**56**)_2_] against ATCC 10213, all the complexes demonstrated weak activity, with a more pronounced effect against ATCC 10213 than against MFBF 11100. [Ga­(L**55**)_2_] exhibited moderate growth inhibition against both ATCC 10213 and MFBF 11100 (IC_90_ 37.28 and 64.42 μM, respectively), and [Ga­(L**56**)_2_] against ATCC 10213 (IC_90_ 79.97 μM). Additionally, Ga­(NO_3_)_3_ had an IC_90_ range of 155.2–162.6 μM toward the tested isolates, suggesting that the observed activities could be related to the synergistic effect of the ligand with gallium ion. In contrast to their iron analogues, all the gallium complexes were active showing a significant improvement in the biological activity by chelation with Ga­(III), except for [Ga­(L**55**)_2_] and its ligand. Furthermore, gallium complexes derived from ligands with a methoxy group (EDG) were more effective than those with a hydroxy group (EDG) (Figure 17), highlighting the importance of the ligand′s substituent in enhancing antifungal activity.

**Figure 17:**
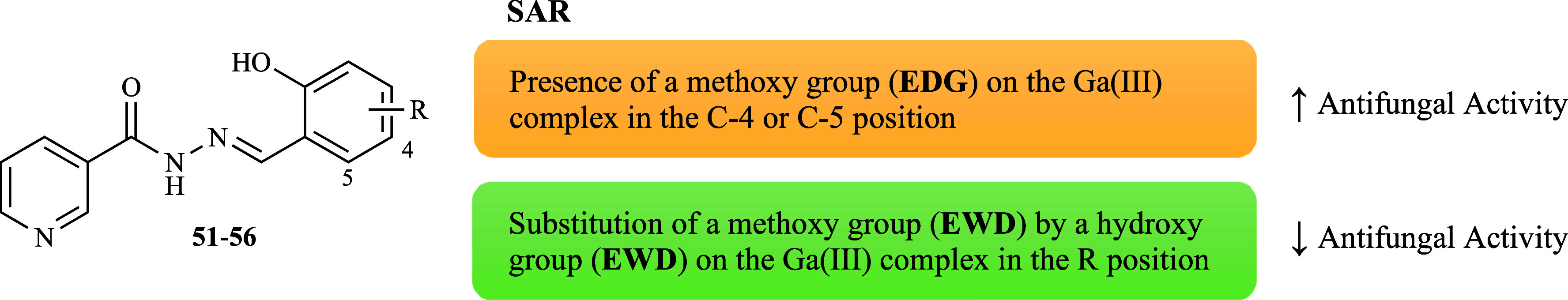
Putative SAR for the antifungal activity of gallium complexes derived from ligands **51**–**56**.

The anti-*C. albicans* ATCC 10231 properties of a series of 2-acetylpyridine-derived thiosemicarbazones (**59**–**65**, Figure 18) which were subsequently complexed with gallium [Ga­(L**59**–**61**, **63**)_2_]­NO_3_ and [Ga­(L**62**)_2_]­NO_3_·3H_2_O were assayed.^89^ The data showed that all the compounds were highly effective against the reference yeasts. Compounds **61** and **62** were found to be active at the lowest concentrations (MIC 10.7 μM), followed by compounds **60** and **59**, showing that the presence of a nitro group has a more favorable effect on activity than a halogen group; however, no change was observed for an iodine substituent (Figure 19). The coordination of Ga­(III) resulted in an improvement of the anti-*C. albicans* effect with different conclusions from the ligands. The introduction of halogen substituents (EWG) was more beneficial in increasing the activity than a nitro substituent (EWG) (Figure 19). Nonetheless, [Ga­(L**63**)_2_]­NO_3_ displayed a MIC value very similar to that of [Ga­(L**60**)_2_]­NO_3_ (7.6 and 7.9 μM, respectively), suggesting that a methyl group (EDG) is as beneficial as a fluorine or iodine group (Figure 19). Notably, [Ga­(L**60**–**61**)_2_]­NO_3_ (MIC 5.9 and 6.2 μM, respectively) were comparable to or more active than FLZ (MIC 6.2 μM). This indicates that gallium complexes with these thiosemicarbazones have the potential to be promising alternatives to first-line antifungal drugs, offering high efficacy in combination with the advantages of metal coordination. The complexes also exhibited notable cytotoxicity against T98G and U87 glioblastoma cells, as well as MCF-7 breast cancer cells, exceeding the cytotoxicity of etoposide, a chemotherapy drug commonly employed for treating various cancers. [Ga­(L**60**)_2_]­NO_3_ displayed greater activity than its corresponding ligand against MCF-7 cells, while [Ga­(L**59**, **61**)_2_]­NO_3_ and [Ga­(L**62**)_2_]­NO_3_·3H_2_O exhibited enhanced activity compared to their ligands against T98G cells. To further evaluate the safety profile of these complexes, their hemolytic activity was assessed by measuring the hemoglobin release, which serves as an indicator of membrane damage. Per physiological conditions (pH 7.4), all of the studied complexes exhibited minimal hemolytic activity (IC_50_ > 10^–5^ mol/L), indicating a negligible disturbance of the red blood cells’ membrane. These findings indicate that the gallium complexes possess a favorable therapeutic index, whereby the potential for damage to healthy cells is minimized while effective targeting of cancer cells is maintained.

**Figure 18:**
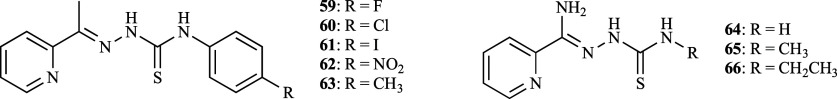
Structure of metal-free ligands **59**–**66**.

**Figure 19:**
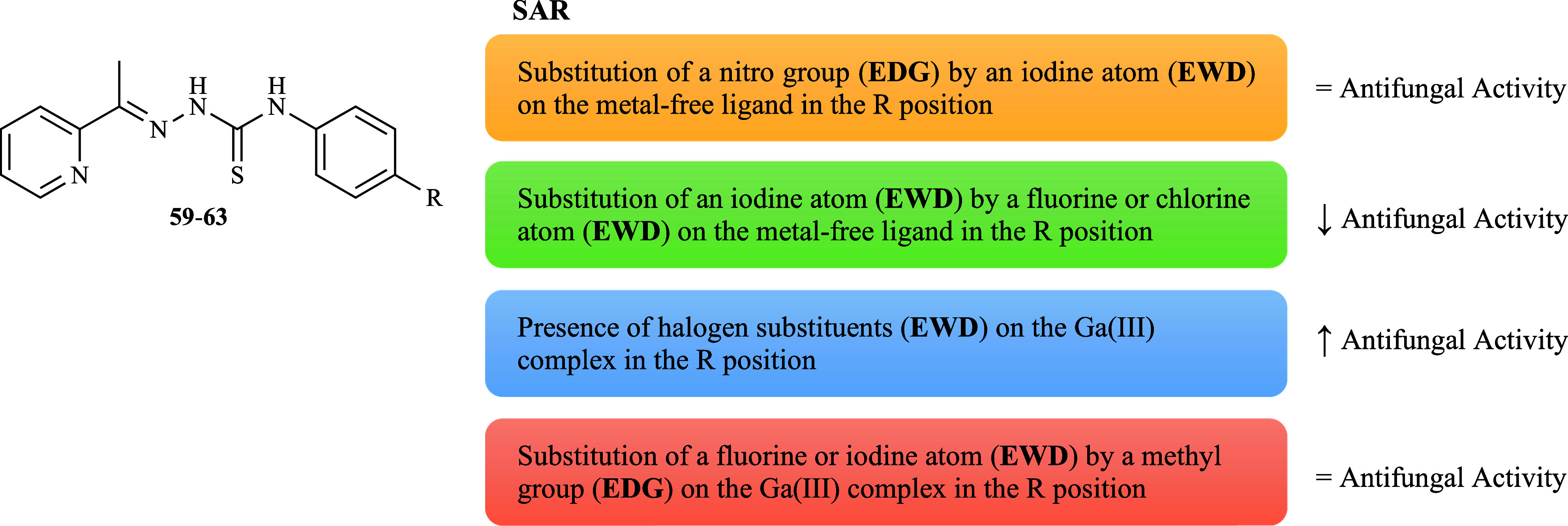
Putative SAR for the antifungal activity of ligands **59**–**63** and their gallium complexes.

A series of 2-pyridineformamide-derived thiosemicarbazones (**64**–**66**, Figure 18) and their corresponding nitrate-gallium complexes [Ga­(**64**–**66**)_2_]­NO_3_ were tested against a range of reference strains and clinical isolates of *Cryptococcus* spp.^90^ Overall, the tested compounds and complexes showed fungistatic activity against *C. neoformans* and *Cryptococcus gattii*. Among the free ligands, compound **64** was identified as the most active (minimum concentration that inhibited 90% growth, MIC_90_ 26.26–52.53 μM), displaying similar activity to FLZ (MIC_90_ 52.24 μM). Subsequently, compound **65** presented higher activity against the majority of the strains (MIC_90_ 51.99–208.0 μM), yet this was comparable to the activity of compound **66**. Notably, *C. gattii* LMM 818 was more sensitive to compound **65** (MIC_90_ 51.99 μM) than to compound **64** (MIC_90_ 52.53 μM). The presence of alkyl groups influences the MIC_90_ values, i.e. the introduction of a methyl-substituent (EDG) led to a decrease in the anticryptococcal activity and an ethyl-substituent (EDG) to a total loss of activity (Figure 20). Coordination to Ga­(III) considerably improved the activity in all cases. In contrast to their ligands, [Ga­(**65**)_2_]­NO_3_ was the most potent (MIC_90_ 2.009–4.018 μM) and showed significantly higher activity than **FLZ** (MIC_90_ 13.06–52.24 μM) against all tested strains, followed by [Ga­(**66**)_2_]­NO_3_ and [Ga­(**64**)_2_]­NO_3_ (MIC_90_ 13.07–26.14 and 13.06–26.13 μM, respectively). The complexes derived from compounds **64** and **66** were more active or as active as **FLZ** against several isolates. This enhanced activity underscores the potential of gallium complexes in therapeutic applications and their efficacy in comparison to existing drugs. It can be explained by the synergism of gallium and thiosemicarbazones, since Ga­(NO_3_)_3_ revealed an anticryptococcal effect against the ten strains and that activity becomes similar to that of FLZ upon complexation with Ga­(III).

**Figure 20:**
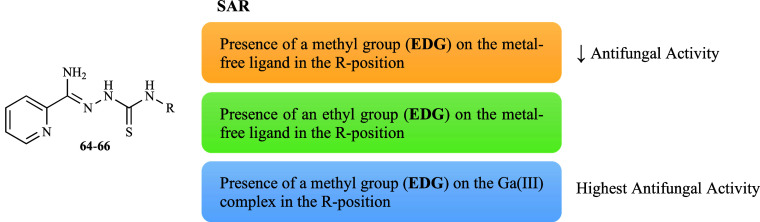
Putative SAR for the antifungal activity of ligands **64**–**66** and their gallium complexes.

To summarize the most relevant data, Table 1 presents the most promising examples discussed. While a generalized SAR could not be established, the data emphasize the pivotal role of specific structural features, such as Schiff bases and electron-withdrawing substituents (e.g., chlorine), along with the choice of metal ion and co-ligand(s), in profoundly influencing antifungal activity and selectivity. Notably, the specific binding affinity between metal and ligand(s) and its influence on biological activity and pharmacokinetics remains underexplored, representing a significant gap in our understanding of the relationship between coordination chemistry and efficacy. These findings highlight the significance of molecular design in enhancing antifungal efficacy and encourage the necessity for further investigation into structure–property relationships and the function of metal–ligand interactions to facilitate the advancement of metal-based compounds. The majority of research has concentrated on the *in vitro* efficacy of metal-based antifungal compounds, with less attention devoted to their *in vivo* behavior. The complexes under discussion in this article are drawn from recent studies, and as a consequence, there is currently no established *in vivo* evidence regarding their antifungal potential. It is therefore imperative that further research is required to assess the overall therapeutic efficacy of these complexes in animal models, which represents a crucial step before advancing to clinical trials.

**Table 1:**
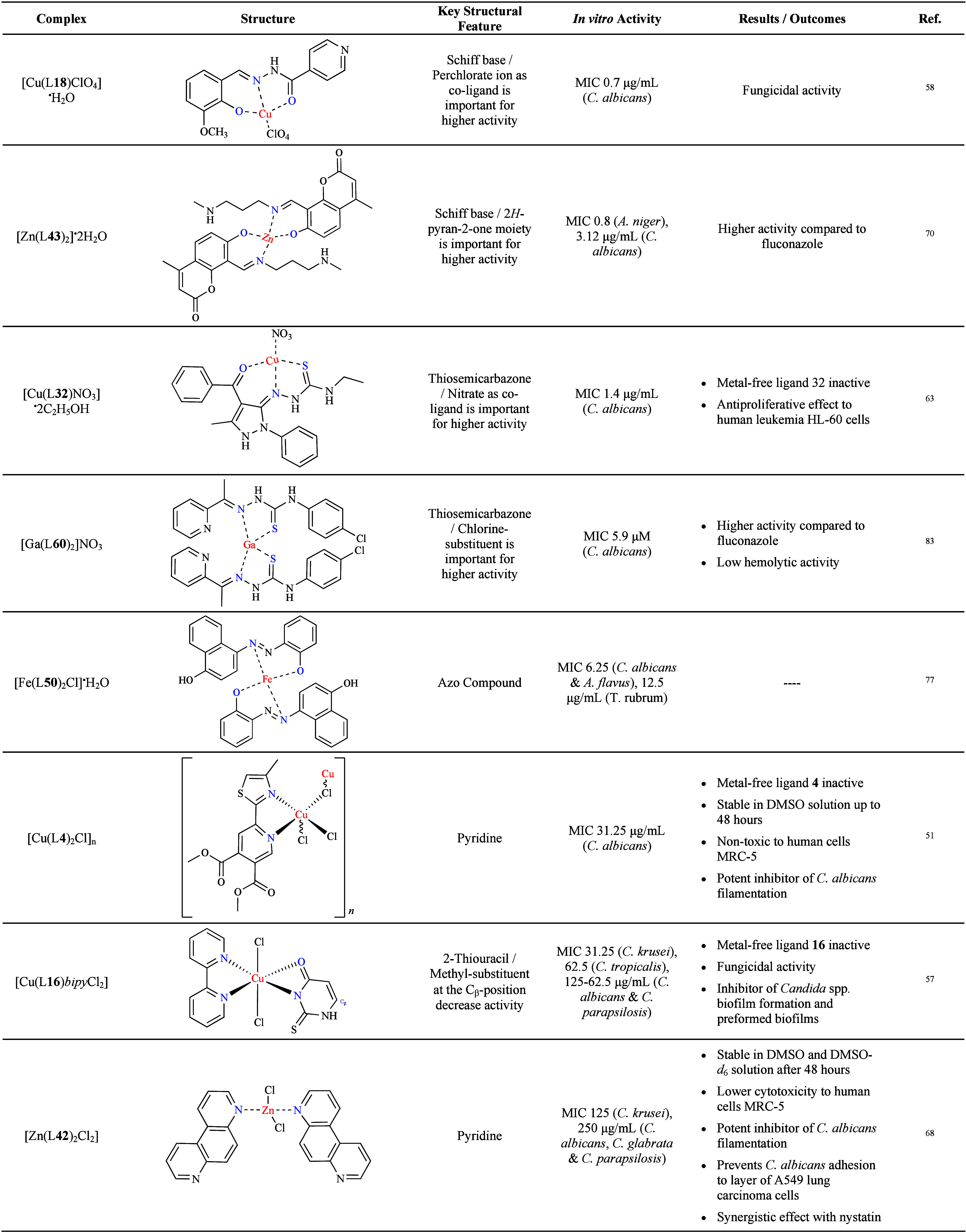
Overview of Key Features of the Most Promising Antifungal Metal Complexes^a^

a MIC: minimum inhibitory concentration; DMSO: dimethyl sulfoxide; DMSO-*d*_6_: deuterated dimethyl sulfoxide; *C. albicans*: *Candida albicans*; *C. krusei*: *Candida krusei*; *C. parapsilosis*; *A. niger*: *Aspergillus niger*; *T. rubrum*: *Trichophyton rubrum*; bipy: bipyridine.

## 3 Conclusions

Antifungal resistance to traditional antifungal agents is an emerging problem worldwide and, as a result, there is an urgent need to discover and develop new and effective drugs. In this regard, metal-based antifungal compounds can be used to design innovative drugs with unique properties. The coordination with metal ions can enhance antifungal activity via different modes of action and thus potentially circumvent resistance.

In this perspective, the antifungal activity of a selected number of organic molecules and their respective metal complexes was explored. From the analysis of the reported examples, it was often shown that metal complexes outperform metal-free ligands in inhibiting fungal growth, where, in certain instances, these complexes were found to be potently effective when compared to their inactive ligands. This corroborates the idea that the coordination of ligands with metal ions can indeed enhance their activities. Overall, the majority of the discussed examples demonstrated considerable activity against drug-resistant strains, such as *C. krusei*, *A. niger*, and mostly *C. albicans*. Although the precise mechanism of action remains to be fully investigated, the antifungal activity of some of these complexes could be related to the synergistic effect between ligand and metal ion or their interference with *C. albicans*′s virulence factors at subinhibitory concentration(s). Undeniably, the results obtained from different examples were consistent with prior research that indicated the ability of metal chelators to disrupt the dimorphic transition and biofilm formation of *C. albicans*, which is frequently associated with its pathogenicity and resistance. Another noteworthy observation demonstrated by some complexes was the fungicidal activity against *Candida* species and the relatively non-toxic or low toxicity to healthy human fibroblast cells. These two findings are extremely important as the ideal antifungal compound should selectively kill fungal cells without harming human cells to prevent or reduce side effects.

Although different methods were used to determine the activity and no general structural features can be identified for these examples or a common SAR can be established, it can be observed that the antifungal activity depends on the nature of the metal ion. In most cases, Zn­(II) complexes were more active than the corresponding Cu­(II) complexes. Due to the limited examples of Fe­(III) and Ga­(III) complexes, conclusive comparatives cannot be made at this time; it is pertinent and worthwhile to perform more studies and explore the potential of other metal­(III) complexes. Nonetheless, the antifungal data underscore the significance of electron-withdrawing substituents in contrast to electron-donating substituents and the distinctive structure characteristic of Schiff bases in metal-based compounds.

In conclusion, the idea that metal-based drugs could be a key approach to overcoming antifungal resistance has been reinforced by the analysis of a selected number of recent antifungal metal complexes. Nevertheless, there are also some limitations associated with metal-based treatment. Many metals can be toxic at certain doses and some metals are vulnerable to oxidation or hydrolysis, which can affect their therapeutic efficacy or can interact with a wide range of biological molecules, leading to potential off-target effects. Therefore, *in vivo* and cytotoxicity studies, and more in-depth assays to identify the mechanisms of action are urgently needed, as the true efficacy of the complexes cannot be fully demonstrated without these studies. Not least because metal-based antimicrobial agents are distinguished by their robust affinity for binding to nucleic acids and proteins, which underlies their bactericidal and fungicidal mechanisms. In the future, it would be relevant and interesting to explore the coordination of various metal ions with the same ligand in the development of the complexes, to further extend the studies of SAR studies to facilitate optimization.

## Abbreviations Used

ATCC: American Type Culture Collection
CTZ: cetoconazole
DFT: density functional theory
DMSO: dimethyl sufoxide
DMSO-*d*_6_: deuterated DMSO
DHA: 2,3-dihydroxybenzoic acid
ECZ: econazole
EDG: electron-donating group
EWG: electron-withdrawing group
FLZ: fluconazole
IC_50_: drug concentration required for 50% inhibition
IC_90_: drug concentration required for 90% inhibition
IR: infrared
KTZ: ketoconazole
MIC: minimum inhibitory concentration
MIC_90_: minimum concentration that inhibited 90% growth
MTCC: Microbial Type Culture Collection and Gene Bank
NMR: Nuclear Magnetic Resonance
PPL: poly­(l-lysine hydrochloride)
SAR: structure–activity relationship
TCZ: tioconazole
TGA: thermogravimetric analysis
UV–vis: ultraviolet–visible
VCZ: voriconazole
